# Pan-HDAC Inhibitors Promote Tau Aggregation by Increasing the Level of Acetylated Tau

**DOI:** 10.3390/ijms20174283

**Published:** 2019-09-01

**Authors:** Hyeanjeong Jeong, Seulgi Shin, Jun-Seok Lee, Soo Hyun Lee, Ja-Hyun Baik, Sungsu Lim, Yun Kyung Kim

**Affiliations:** 1 Convergence Research Center for Diagnosis, Treatment and Care System of Dementia, Brain Science Institute, Korea Institute of Science and Technology (KIST), Seoul 02792, Korea; 2 Department of Life Sciences, Korea University, Seoul 02841, Korea; 3 Division of Bio-Medical Science & Technology, University of Science and Technology (UST), Daejeon 34113, Korea; 4 Molecular Recognition Research Center, Korea Institute of Science and Technology (KIST), Seoul 02792, Korea; 5 Center for Biomicrosystems, Korea Institute of Science and Technology (KIST), Seoul 02792, Korea

**Keywords:** histone deacetylase inhibitor, tau acetylation, tau aggregation, Alzheimer’s disease

## Abstract

Epigenetic remodeling via histone acetylation has become a popular therapeutic strategy to treat Alzheimer’s disease (AD). In particular, histone deacetylase (HDAC) inhibitors including M344 and SAHA have been elucidated to be new drug candidates for AD, improving cognitive abilities impaired in AD mouse models. Although emerged as a promising target for AD, most of the HDAC inhibitors are poorly selective and could cause unwanted side effects. Here we show that tau is one of the cytosolic substrates of HDAC and the treatment of HDAC inhibitors such as Scriptaid, M344, BML281, and SAHA could increase the level of acetylated tau, resulting in the activation of tau pathology.

## 1. Introduction

Alzheimer’s disease (AD) is a chronic neurodegenerative disorder that characterized by extracellular deposits of amyloid plaques and neuronal deposits of tau aggregates composed of hyperphosphorylated tau [1,2]. Over the last few decades, a number of compounds, designed to reduce the formation of amyloid plaques or to enhance their clearance, have failed in clinical trials. Since the causes of AD are still unknown, diverse targets are being applied for anti-Alzheimer’s drug discovery. Among the diverse, epigenetic regulation has been proposed to be a new promising therapeutic strategy for neurological disorders, particularly for AD [3]. In aged animal models, decreased levels of histone acetylation have been observed in the hippocampus and cerebral cortex [4,5]. Such changes could contribute to the development of neurodegeneration by down-regulating genes, which are critical for learning and memory. A number of recent studies have showed that histone deacetylase (HDAC) inhibitors exhibit neuro-protective properties, rescuing learning and memory abilities impaired in AD animal models [4,6,7,8,9,10,11]. Accordingly, HDAC inhibition has emerged as an alternative therapeutic strategy in AD treatment.

Histone deacetylases are divided into four classes. Class I, II, and IV contain the classic HDAC enzymes, and Class III contains the sirtuin enzymes, which require NAD+ as a cofactor [12,13]. In 2009, Francis et al. proposed that epigenetic alteration by HDAC inhibition could be a therapeutic target to prevent AD progression [7]. In their study, trichostatin A, a HDAC inhibitor, rescued fear memory impaired in APP/PS1 mice by increasing the acetylation of histone H4. In 2010, Peleg et al. reported that SAHA, a HDAC inhibitor, rescued age-dependent memory impairment in old mice by increasing the acetylation of histone H4 [4]. In 2017, Volmar et al. also suggested M344, a HDAC inhibitor, to be a promising drug candidate for AD [6]. In their study, M344 prevented cognitive declines in an AD animal model by down-regulating AD-related genes that contribute to APP processing and tau phosphorylation. Accumulating studies have supported that other HDAC inhibitors including valproic acid, 4-phenylbutyrate, MPT0G211, and nicotinamide presented similar therapeutic effects in AD animal models [8,10,14,15,16]. However, histone is not the only substrate of HDACs. A variety of non-histone substrates of HDACs exist in nuclei and cytosols [17,18]. Most HDAC inhibitors listed above are poorly selective and could cause unwanted side effects by acetylating non-histone proteins.

In fact, several studies have showed that tau is a direct substrate of HDAC. In 2011, Cohen et al. reported that trichostatin A, a pan-HDAC inhibitor, increased tau acetylation [19]. In 2014, Noack et al. also reported that tubastatin A, a HDAC6 inhibitor, increased acetylated tau [20]. Tau is a neuron-specific microtubule-binding protein that stabilizes microtubules [21,22,23]. When pathologically modified, tau dissociates from microtubules and becomes insoluble aggregates [24,25,26,27,28]. Although hyperphosphorylation has been considered to be the major modification of tau, initiating tau pathology [2,29], recent studies have demonstrated that tau acetylation is also strongly associated with tau pathology [19,30,31]. Elevated levels of acetylated tau have been observed in AD patients [19,30], and acetylated tau has been colocalized with insoluble tau aggregates in the brain of AD animal models [19,32,33]. Biochemical studies have identified that tau acetylation slows down tau turnover, inhibiting proteasomal degradation [20,34]. In a result, acetylated tau accumulates, activating tau aggregation [19,30,34,35] and increased acetylated tau levels in the brain of tau transgenic mouse models are enough to cause an acetylation-mediated tau pathological cascade [32,33]. Due to the pathological implication, tau acetylation should be carefully evaluated in HDAC-targeting drug discovery.

In this study, we collected 34 commercially available inhibitors of histone deacetylases (HDACs, SIRTs) and histone acetyl transferases (HATs) and evaluated the effect on tau acetylation and aggregation. Among the tested, pan-HDAC inhibitors (Scriptaid, M344, BML281, and SAHA) markedly induced intracellular tau aggregation. All the selected HDAC inhibitors increased the acetylation of tau at the residue K280 strongly as well as its representative cytosolic and nucleic substrates, tubulin and histone.

## 2. Results

### 2.1. Evaluation of HDAC Modulators on Tau-BiFC Sensor

**Figure 1:**
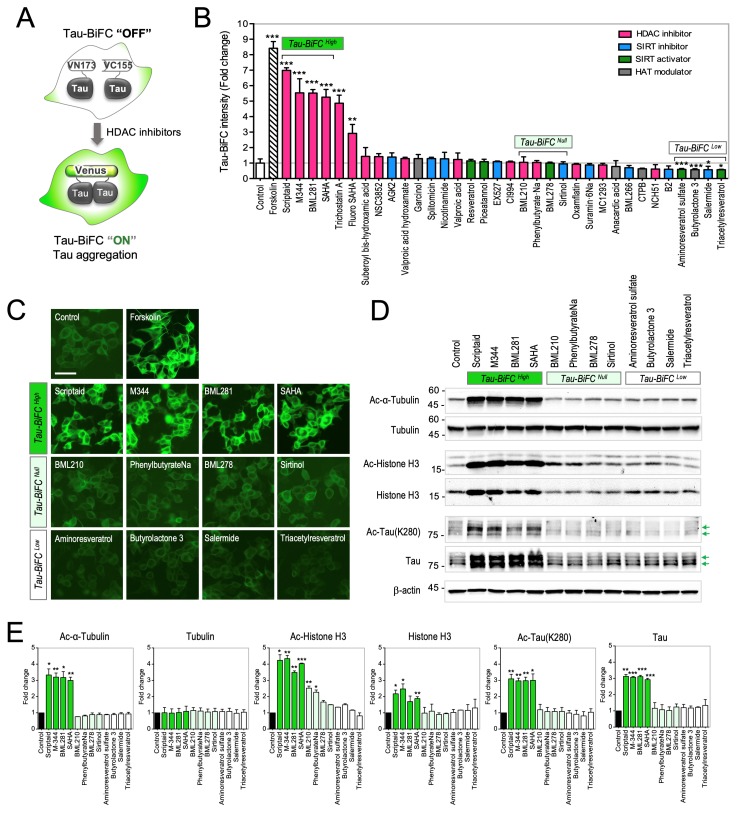
Evaluation of HDAC modulators on tau-BiFC sensor. (**A**) Illustration of tau-BiFC cell system. Tau-BiFC cell expresses full-length tau conjugated with the non-fluorescent N- or C-terminal fragments of Venus fluorescence protein. When tau assembles together, Venus fluorescence turns on. (**B**) Screening of 34 compounds in tau-BiFC cells. Tau-BiFC cells were treated with 34 library compounds at 3 µM for 48 h. BiFC fluorescence intensities were quantified using Harmony 3.1 software. The BiFC intensities of compound-treated groups were normalized to that of control. Pink, HDAC inhibitor; blue, SIRT inhibitor; green, SIRT activator; and grey, HAT modulator. Data represent the mean ± S.D. of four independent experiments. * *p* < 0.05. ** p < 0.01, *** *p* < 0.001 compared to control. (**C**) BiFC fluorescence images of tau-BiFC cells treated with tau-BiFC^High^, tau-BiFC^Null^, and tau-BiFC^Low^ groups. Tau-BiFC cells were treated with compounds of tau-BiFC^High^ (Scriptaid, M344, BML281, and SAHA), tau-BiFC^Null^ (BML210, PhenylbutyrateNa, BML278, and Sirtinol), and tau-BiFC^Low^ (Aminoresveratrol sulfate, Butyrolactone 3, Salermide, and Triacetylresveratrol) groups at 3 µM for 36 h, and imaged. Scale bar, 50 µm. (**D**) Immunoblot analysis of acetylated and total of tubulin, histone H3, and tau with anti-ac-α-tubulin, anti-α/β-tubulin, anti-ac-histone H3, anti-histone H3, anti-ac-Tau(K280), and anti-Tau5 antibodies. β-actin was used as a loading control. Green arrows indicate Tau-VN173 and Tau-VC155. (**E**) Quantification of acetylation and total expression levels of tubulin, histone H3, and tau. The relative amounts of acetylated and total tubulin, histone H3, and tau were quantified by Image J. Data represent the mean ± S.D. of replicate experiments. * *p* < 0.05. ** *p* < 0.01, *** *p* < 0.001.

**Figure 2:**
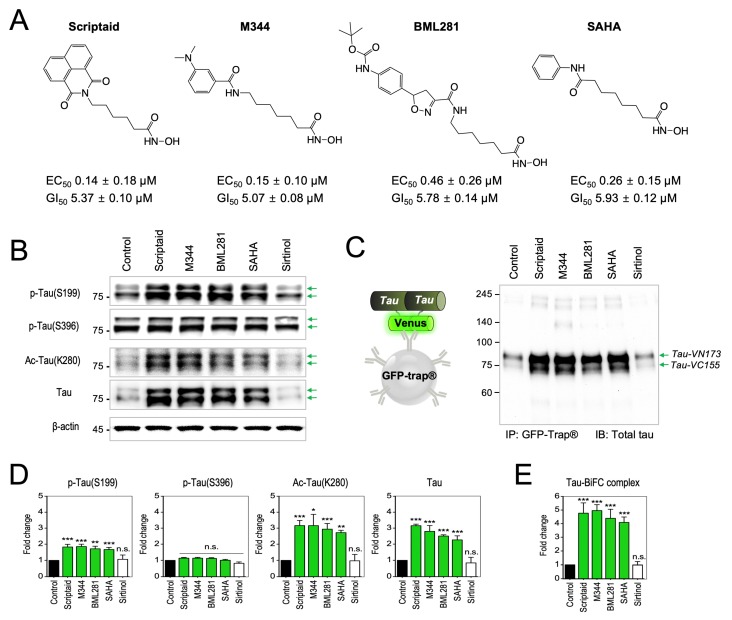
Activation of tau pathology by the treatment of pan-HDAC inhibitors. (**A**) Structures of Scriptaid, M344, BML281, and SAHA with EC_50_ and GI_50_ values. Tau-BiFC cells were incubated with pan-HDAC inhibitors at various concentrations (0.1, 0.3, 1, 3, 10, 30 µM) for 36 h. A Prism’s non-linear regression analysis was used to measure the EC_50_ and GI_50_ values. (**B**) Immunoblot analysis of phosphorylated, acetylated and total tau with anti-p-Tau(S199), anti-p-Tau(S396), anti-ac-Tau(K280), and Tau5 antibodies. For the immunoblot analysis, tau-BiFC cells were treated with Scriptaid, M344, BML281, SAHA, or Sirtinol at 3 µM for 36 h. Green arrows indicate two parts of tau-conjugated BiFC compartments, Tau-VN173 and Tau-VC155. Anti-β-actin was used as a loading control. (**C**) Immunoblot analysis of total tau in GFP-trap fractions with Tau5 antibody. Tau-BiFC cells were treated with Scriptaid, M344, BML281, SAHA, or Sirtinol at 3 µM for 36 h. The cells were lysed and then, incubated with GFP-trap beads to pull down the paired tau-BiFC complexes. (D,E) Quantification of phosphorylated, acetylated, and total tau in total cell lysates (**D**) and total tau in GFP-trap fractions (**E**). The relative amounts of phosphorylated, acetylated and total tau were quantified by Image J. Data represent the mean ± S.D. of replicate experiments. * *p* < 0.05. ** *p* < 0.01, *** *p* < 0.001.

For the comprehensive evaluation of HDAC inhibitors, we collected 30 commercially available modulators of histone deacetylases (HDACs, SIRTs), together with 4 modulators of histone acetyl transferases (HATs). Then, the compound effects on tau aggregation were evaluated by using a tau aggregation sensor, named tau-BiFC [36]. As a fluorescence turn-on sensor, tau-BiFC fluorescence turns on only when tau assembles together (Figure 1A). Tau-BiFC fluorescence directly represents the level of tau assembly in a cell, from soluble dimers to insoluble aggregates [36,37,38,39,40]. Forskolin was used as a positive control [36,41]. Among tested, 6 HDAC inhibitors, Scriptaid, M344, BML281, SAHA, Trichostatin A, and Fluoro-SAHA, induced tau-BiFC fluorescence noticeably by showing more than 3-fold increase at 3 µM concentration (Figure 1B). In particular, Scriptaid, M344, BML281, and SAHA, HDAC inhibitors containing an aliphatic hydroxyamide linker acid, showed the strongest tau-BiFC fluorescence responses (Figure 1C and Figure 2A).

To investigate whether a tau-BiFC response correlates with the substrate specificity of the HDAC inhibitors, the compounds were categorized into three groups. Scriptaid, M344, BML281, and SAHA were grouped as Tau-BiFC^High^. BML210, PhenylbutyrateNa, BML278, and Sirtinol, which did not induce any change in the tau-BiFC response, were grouped as Tau-BiFC^Null^. Aminoresveratrol sulfate, Butyrolactone 3, Salermide, and Triacetylresveratrol, which showed slightly lower BiFC intensities than that of control, were grouped as Tau-BiFC^Low^ (Figure 1C). Immunoblot analysis was followed to evaluate acetylation levels of α-tubulin, a cytoplasmic substrate of HDACs, and histone H3, a nuclear substrate of HDACs [42,43]. The Tau-BiFC^High^ group strikingly elevated both α-tubulin acetylation and histone H3 acetylation. The acetylation levels of α-tubulin increased over 3.0- up to 3.3-fold, and the acetylation levels of histone H3 increased over 3.5- up to 4.3-fold. In comparison, Tau-BiFC^Null^ and Tau-BiFC^Low^ groups did not show noticeable changes in α-tubulin acetylation (Figure 1D,E). In the Tau-BiFC^Null^ group, BML210 and PhenylbutyrateNa slightly increased histone acetylation by showing 2.5- and 2.3-fold increases. The results indicate that Scriptaid, M344, BML281, and SAHA are pan-HDAC inhibitors, which strongly inhibit both cytoplasmic and nuclear HDACs. As a cytosolic substrate of HDACs, tau was also strongly acetylated by pan-HDAC inhibitors. Similar to the increased level of acetylated tubulin, Tau(K280) acetylation increased almost 3-fold by the treatment of the pan-HDAC inhibitors. Different from acetylated tubulin, acetylated tau seems accumulated in the cells, increasing the amount of total tau.

### 2.2. Activation of Tau Pathology by the Treatment of Pan-HDAC Inhibitors

Next, we scrutinized tau pathology activated by Scriptaid, M344, BML281, and SAHA. Dose-dependent analysis indicated that Scriptaid, M344, BML281, and SAHA have sub-micromolar EC_50_ values in activating tau-BiFC fluorescence (Scriptaid, EC_50_ = 0.14 ± 0.18; M344, EC_50_ = 0.15 ± 0.10; BML281, EC_50_ = 0.46 ± 0.26; and SAHA, EC_50_ = 0.26 ± 0.15 μM; Figure 2A and Figure S1). 50% of maximal inhibition of cell proliferation (GI_50_) values were determined 48 h after the treatment to tau-BiFC cells (Scriptaid, GI_50_ = 5.37± 0.10; M344, GI_50_ = 5.07 ± 0.08; BML281, GI_50_ = 5.78 ± 0.14; and SAHA, GI_50_ = 5.93 ± 0.12 μM). It is possible that other HDAC inhibitors could increase tau-BiFC response at higher concentrations. However, Sirtinol, a SIRT inhibitor, which did not induce tau-BiFC response up to 30 μM, was used as a negative control.

For immunoblot analysis, tau-BiFC cells were treated with each compound for 36 h and cell lysates were prepared. S199 and S396 are the representative phosphorylation sites of tau phosphorylated by GSK-3β [44]. While the levels of phospho-Ser396 (pS396) did not change, the levels of phospho-Ser199 (pS199) increased slightly upon the treatment of the selected HDAC inhibitors, by showing 1.7~1.9-fold increases (Figure 2B). Compare to the phosphorylation levels, tau acetylation (Ac-K280) increased more strikingly by showing 2.7~3.2-fold increases upon the treatment of Scriptaid, M344, BML281, or SAHA. Total tau levels were also elevated over 2.7- up to 3.1-fold. (Figure 2B,D). The elevated levels of total tau closely matched with the levels of acetylated tau than that of phosphorylated tau. To evaluated the level of tau aggregation, matured tau-BiFC complexes in each cell lysates were enriched by using GFP-trap^®^ (Figure 2C) [45]. GFP-trap^®^ captures only paired BiFC complexes, not N- or C-terminal fragments of Venus. GFP-trap^®^-captured tau-BiFC complexes directly indicate the level of tau aggregation from soluble dimers to insoluble aggregates. Upon the treatment of Scriptaid, M344, BML281, or SAHA, GFP-trap^®^-captured tau-BiFC complexes were significantly increased by showing 4.0~4.9-fold increases (Figure 2C,E). Our results correspond to the previous studies, suggesting that tau acetylation slows tau turnover by inhibiting proteasomal degradation and leads to the accumulation of tau [20,33,34]. Especially, tau acetylation at K280/K281 is known to be critical in fibrillar tau aggregation [19,46]. Taken these together, our results suggest that tau acetylation increased by pan-HDAC inhibitors could lead pathological tau accumulation and aggregation.

### 2.3. Increase of Tau Acetylation by the Inhibition of Cytoplasmic HDAC6

**Figure 3:**
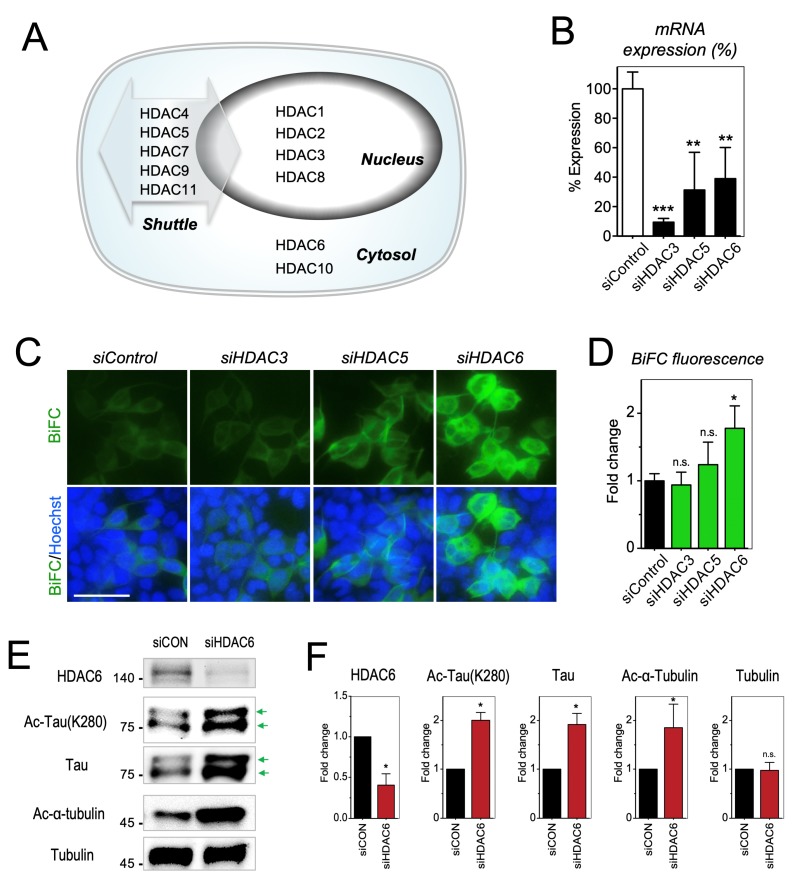
Increase of tau acetylation by the inhibition of cytosolic HDAC6. (**A**) Cellular localization of HDAC enzymes. A total of 11 HDACs belonging to three classes were mainly distributed in nucleus, cytosol, and both nucleus and cytosol (shuttle). (**B**) mRNA expression levels of HDACs in siHDACs-transfected tau-BiFC cells. Tau-BiFC cells were transfected with HDAC3, HDAC5, or HDAC6 siRNA. After 72 h of transfection, total mRNA was extracted and then cDNA synthesized from total mRNA was analyzed by q-PCR with HDAC3, HDAC5, or HDAC6 specific primers. (**C**) BiFC fluorescence images of tau-BiFC cells transfected with siRNA targeting HDAC3, HDAC5, or HDAC6. Nuclei were counterstained with Hoechst. Scale bar, 50 µm. (**D**) Quantification of BiFC fluorescence intensities. (**E**) Immunoblot analysis of HDAC6, acetylated and total tubulin, and acetylated, phosphorylated, and total tau with anti-HDAC6, anti-ac-α-tubulin, anti-α/β-tubulin, anti-ac-Tau(K280), anti-p-Tau(S199), and Tau5 antibodies. Green arrows indicate Tau-VN173 and Tau-VC155. (**F**) Quantification of HDAC6, acetylated and total tubulin, and acetylated, phosphorylated, and total tau levels. (**C**,**E**,**G**) Data represent the mean ± S.D. of replicate experiments. * *p* < 0.05. ** *p* < 0.01, *** *p* < 0.001.

Scriptaid, M344, BML281, and SAHA are pan-HDAC inhibitors that are known to inhibit both nuclear and cytoplasmic HDACs [6,47,48,49,50] (Table 1). Depending on the subcellular localization, HDAC enzymes can be divided into three classes [51]. Class I (HDAC 1, 2, 3, and 8) are primarily located in nucleus. Class IIa (HDAC 4, 5, 7, and 9) and class IV (HDAC 11) shuttle between nucleus and cytoplasm. Class IIb (HDAC 6 and 10) are primarily found in cytosol (Figure 3A). To investigate HDAC subclasses that affect tau acetylation, we prepared siRNAs for HDAC3, HDAC5, and HDAC6, representing each subclass. Tau-BiFC cells were transfected with siRNAs against HDAC3, HDAC5, or HDAC6. After 72 h, mRNA expression of each of HDACs was evaluated (Figure 3B). By siRNA transfection, mRNA expression reduced 90% in HDAC3, 68% in HDAC5, and 61% in HDAC6, compared to that of scrambled control (siControl). As expected, HDAC6 knockdown led to 1.8-fold increase in tau-BiFC fluorescence, while HDAC3 knock-down did not affect tau-BiFC response. (Figure 3C,D). HDAC5 knock-down increased tau-BiFC response slightly, but the increase was not significant. Immunoblot analysis was followed to investigate tau acetylation levels upon HDAC6 knock-down (Figure 3E). Comparable to the reduced mRNA levels, HDAC6 protein expression decreased by 60% by siRNA transfection. In result, acetylated α-tubulin increased 1.9-fold and acetylated tau increased 2-fold. Similar to the treatment of the pan-HDAC inhibitors, HDAC6 knockdown increased total tau, but not total tubulin (Figure 3E,F). Although the role of HDAC6 in tau pathology is still controversial [20,52,53], our result shows that tau is constantly deacetylated by cytoplasmic HDACs. Therefore, inhibition or knock-down of cytoplasmic HDACs increases tau acetylation, leading to pathological tau accumulation.

## 3. Discussion

Histone acetylation play a critical role in memory formation and synaptic elasticities in hippocampus, and alterations of histone acetylation were observed in AD mouse models and AD patients [4,6,7,54,55,56]. Accordingly, histone deacetylases (HDACs) emerged as a new therapeutic target for AD. Accumulating studies also showed the therapeutic potential of HDAC inhibitors (M344, SAHA, and Trichostatin A), of which administration rescued learning and memory abilities impaired in an AD mouse model [4,6,7]. However, HDACs acetylate not only histone, but also a variety of non-histone proteins in nuclei and cytosols [17,18]. Since most HDAC inhibitors are poorly selective, the treatment would cause unwanted side effects. Here, we show that tau is one of the cytosolic substrates of HDACs and the treatment of pan-HDAC inhibitors (Scriptaid, M344, BML281, and SAHA) significantly increase tau acetylation and aggregation.

Tau acetylation is strongly associated with AD pathology [19,30,31,32,33]. Biochemically, acetylation of tau inhibits its degradation, leading to pathological tau accumulation [20,33,34]. In vitro study has also demonstrated that tau acetylation at residues K280/K281 promote the formation of fibrillar tau aggregates [19,46]. Acetylated tau has been colocalized with insoluble tau aggregates in AD mice models [19,32,33], and elevated tau acetylation has also been observed in the brains of AD patients [19,30,32,33]. Considering the pathological implication in AD, tau acetylation should be carefully evaluated in HDAC-targeted drug development. 

## 4. Materials and Methods

### 4.1. Cell Culture and Compound Screening

HEK293 tau-BiFC cells were maintained in DMEM containing 10% FBS, 100 units/mL penicillin, 100 µg/mL streptomycin, and 100 µg/mL Geneticin (G418, Sigma, St. Louis, MO, USA) at 37 °C in a humidified atmosphere containing 5% CO_2_.

For screening of HDACs, SIRTs, and HATs modulators, tau-BiFC cells were plated on µ-clear 384-well plates. The next day, the cells were treated with 34 library compounds at 3 µM concentration. After 48 h, nuclei were counterstained with 2 µg/mL Hoechst (Invitrogen, Waltham, MA, USA). The entire 384-well plate was automatically imaged by using Operetta^®^ (PerkinElmer, Waltham, MA, USA). High resolution images were acquired by using a Nikon Eclipse inverted microscope (Ti, Nikon, Tokyo, Japan) at 200× magnification. SCREEN-WELL^®^ Epigenetics library was purchased from Enzo Life Sciences Inc. (Farmingdale, NY, USA). Forskolin (Sigma, St. Louis, MO, USA) was used as a positive control.

### 4.2. BiFC-Image Analysis

BiFC fluorescence images were acquired using Operetta and analyzed using Harmony 3.1 software (PerkinElmer, Waltham, MA, USA). All experiments were performed in triplicate. The means and standard deviations (S.D.) of tau-BiFC fluorescence intensities were plotted by using Prism7 software (GraphPad, San Diego, CA, USA). Quantification data was analyzed by Student’s *t-*test. 

### 4.3. Immunoblot Analysis

For the immunoblot assay, tau-BiFC cells grown in a 100 mm culture dish were treated with a total 12 compounds of tau-BiFC^High^ (Scriptaid, M344, BML281, and SAHA), tau-BiFC^Null^ (BML210, PhenylbutyrateNa, BML278, and Sirtinol), and tau-BiFC^Low^ (Aminoresveratrol sulfate, Butyrolactone 3, Salermide, and Triacetylresveratrol) groups (Tocris, Ellisville, MO, USA) at 3 µM for 36 h. Then, cell lysates were prepared by using RIPA lysis buffer containing protease/phosphatase inhibitor cocktail (Sigma), and deacetylase inhibitors (1 µM TSA and 5 mM Nicotinamide). Quantity of 10 μg of each lysate was separated on 7.5% SDS-PAGE gel and transferred to PVDF membrane. Immuno-blot analysis was performed by using Tau5 (1:2000, AbCam, Cambridge, MA, USA), anti-p-Tau(S199) (1:2000, AbCam), anti-p-Tau(Ser396) (1:2000, AbCam), anti-ac-Tau(K280) (1 µg/mL, Anaspec, Fremont, CA, USA), anti-ac-α-Tubulin(K40) (1:1000, AbCam), anti-α/β-Tubulin (1:1000, Cell signaling, Danvers, MA, USA), anti-ac-Histone H3 (1:500, Abcam), anti-Histone H3 (1:2000, Abcam), and anti-HDAC6 (1:1000, Cell Signaling) antibodies. Band intensity was quantified using Image J software (NIH). Quantification data was analyzed by Student’s t-test.

### 4.4. Isolation of Mature Tau-BiFC Complexes Using GFP-Trap^®^

To isolate tau-BiFC complexes, GFP-Trap^®^ (ChromoTek, Munich, Germany) immuno-precipitation was followed by the manufacturer’s instructions. Briefly, tau-BiFC cells were lysed with RIPA lysis buffer containing protease/phosphatase inhibitor cocktail and deacetylase inhibitors (1 µM TSA and 5 mM Nicotinamide). Each lysate (1 mg) was incubated with 50 µL of GFP-Trap^®^ beads at 4 °C overnight. Beads were washed three times with washing buffer (10 mM Tris/Cl pH 7.5, 150 mM NaCl and 0.5 mM EDTA). Bound proteins were then eluted by adding 120 µL of 2× SDS sample buffer with β-mercaptoethanol and boiled at 95 °C for 5 min.

### 4.5. siRNA Transfection and Analysis

For siRNA transfection, HDAC3, HDAC5, and HDAC6 siRNAs were purchased from OriGene Technologies, Inc. (Rockville, MD, USA). Tau-BiFC cells were plated on µ-clear 96-well plate or 12-well culture plate in Opti-MEM (Invitrogen, Waltham, MA, USA) and transfected with each of siRNAs by using Lipofectamine 2000 (Invitrogen). Scrambled siRNA was used as a control. Twelve hours after transfection, the medium was replaced with fresh growth medium (DMEM containing 10% FBS with antibiotics). Seventy-two hours of transfection, siRNA-transfected tau-BiFC cells were used for BiFC-image analysis and immunoblot analysis.

### 4.6. mRNA Extraction and Real-time Quantitative RT-PCR (QPCR) Analysis

For mRNA extraction, tau-BiFC cells were washed twice with PBS and lysed with 500 µL of TRIzol reagent (Life Technologies, Carlsbad, CA, USA) for 15 min at RT with shaking. Reverse transcription of mRNA was conducted with AccuPower^®^ CycleScript RT PreMix (Bioneer Inc, Daejeon, Korea), and real-time quantitative polymerase chain reaction (RT-qRCR) was performed with FAST qPCR Kit Master Mix (Kapa Biosystems, Cape, South Africa). The reaction was amplified, and mRNA quantity was assessed using QuantStudio 3 (Applied Biosystems, CA, USA) according to the manufacturer’s instructions. The relative quantity (RQ) of mRNA was obtained through the comparative threshold cycle (Ct) method and normalized using GAPDH as an endogenous control. Primer sequences were manufactured from Macrogen (Seoul, Korea). Primer sequences for HDAC3, HDAC5, HDAC6, and GAPDH were as follows: (HDAC3 F) 5′-CTGGCTTCTGCTATGTCAAC-3′, (HDAC3 R) 5′-ACATATTCAACGCATTCCCCA-3′, (HDAC5 F) 5′-CGCTGAGAATGGCTTTACTG GC-3′, (HDAC5 R) 5′-GTGTAGAGGCTGAACTGGTTGG-3′, (HDAC6 F) 5′-GCCTCAATCACTGA GACCATCC-3′, (HDAC6 R) 5′-GGTGCCTTCTTGGTGACCAACT-3′, (GAPDH F) 5′-CGCTCTCTG CTCCTCCTGTT-3′, (GAPDH R) 5′-CCATGGTGTCTGAGCGATGT-3′.

## Figures and Tables

**Table 1:** Target HDACs sub-classes of four pan-HDAC inhibitors (Scriptaid, M344, BML281, and SAHA).

HDAC Inhibitor	Target HDACs	References
Scriptaid	Class I (HDAC 1, 2, 3, 8)	[47,48]
	Class IIb (HDAC 6)	
M344	Class I (HDAC 1, 2, 3, 8)	[6]
	Class IIb (HDAC 6, 10)	
BML281	Class I (HDAC 3)	[49]
	Class IIb (HDAC 6)	
SAHA	Class I (HDAC 1, 2, 3, 8)	[50]
	Class IIa (HDAC 4, 5, 7, 9)	
	Class IIb (HDAC 6, 10)	

## Supplementary Materials

Supplementary materials can be found at https://www.mdpi.com/1422-0067/20/17/4283/s1.

## References

[1] Murpy M., LeVine III H. (2010). Alzheimer’s disease and the β-amyloid peptide. J. Alzheimers Dis..

[2] Iqbal K., Liu F., Gong C.X., Grundke-Iqbal I. (2010). Tau in Alzheimer disease and related tauopathies. Curr. Alzheimer Res..

[3] Xu Z., Li H., Jin P. (2012). Epigenetics-based therapeutics for neurodegenerative disorders. Curr. Geriatr. Rep..

[4] Peleg S., Sananbenesi F., Zovoilis A., Burkhardt S., Bahari-Javan S., Agis-Balboa R.C., Cota P., Wittnam J.L., Gogol-Doering A., Opitz L. (2010). Altered histone acetylation is associated with age-dependent memory impairment in mice. Science.

[5] Walker M.P., LaFerla F.M., Oddo S.S., Brewer G.J. (2013). Reversible epigenetic histone modifications and Bdnf expression in neurons with aging and from a mouse model of Alzheimer’s disease. Age.

[6] Volmar C.H., Salah-Uddin H., Janczura K.J., Halley P., Lambert G., Wodrich A., Manoah S., Patel N.H., Sartor G.C., Mehta N. (2017). M344 promotes nonamyloidogenic amyloid precursor protein processing while normalizing Alzheimer’s disease genes and improving memory. Proc. Natl. Acad. Sci. USA.

[7] Francis Y.I., Fà M., Ashraf H., Zhang H., Staniszewski A., Latchman D.S., Arancio O. (2009). Dysregulation of histone acetylation in the APP/PS1 mouse model of Alzheimer’s disease. J. Alzheimers Dis..

[8] Fan S.J., Huang F.I., Liou J.-P., Yang C.R. (2018). The novel histone de acetylase 6 inhibitor, MPT0G211, ameliorates tau phosphorylation and cognitive deficits in an Alzheimer’s disease model. Cell Death Dis..

[9] Ricobaraza A., Cuadrado-Tejedor M., Marco S., Pérez-Otaño I., García-Osta A. (2012). Phenylbutyrate rescues dendritic spine loss associated with memory deficits in a mouse model of Alzheimer disease. Hippocampus.

[10] Green K.N., Steffan J.S., Martinez-Coria H., Sun X., Schreiber S.S., Thompson L.M., LaFerla F.M. (2008). Nicotinamide restores cognition in Alzheimer’s disease transgenic mice via a mechanism involving sirtuin inhibition and selective reduction of Thr231-phosphotau. J. Neurosci..

[11] Selenica M.L., Benner L., Housley S.B., Manchec B., Lee D.C., Nash K.R., Kalin J., Bergman J.A., Kozikowski A., Gordon M.N. (2014). Histone deacetylase 6 inhibition improves memory and reduces total tau levels in a mouse model of tau deposition. Alzheimers Res. Ther..

[12] Ververis K., Karagiannis T.C. (2012). Overview of the classical histone deacetylase enzymes and histone deacetylase inhibitors. ISRN Cell Biol..

[13] Marks P.A. (2010). Histone deacetylase inhibitors: A chemical genetics approach to understanding cellular functions. Biochim. Biophys. Acta (BBA) Gene Regul. Mech..

[14] Qing H., He G., Ly P.T., Fox C.J., Staufenbiel M., Cai F., Zhang Z., Wei S., Sun X., Chen C.-H. (2008). Valproic acid inhibits Aβ production, neuritic plaque formation, and behavioral deficits in Alzheimer’s disease mouse models. J. Exp. Med..

[15] Hu J.P., Xie J.W., Wang C.Y., Wang T., Wang X., Wang S.L., Teng W.P., Wang Z.Y. (2011). Valproate reduces tau phosphorylation via cyclin-dependent kinase 5 and glycogen synthase kinase 3 signaling pathways. Brain Res. Bull..

[16] Ricobaraza A., Cuadrado-Tejedor M., Pérez-Mediavilla A., Frechilla D., Del Río J., García-Osta A. (2009). Phenylbutyrate ameliorates cognitive deficit and reduces tau pathology in an Alzheimer’s disease mouse model. Neuropsychopharmacology.

[17] Dokmanovic M., Clarke C., Marks P.A. (2007). Histone deacetylase inhibitors: Overview and perspectives. Mol. Cancer Res..

[18] Yao Y.-L., Yang W.M. (2010). Beyond histone and deacetylase: An overview of cytoplasmic histone deacetylases and their nonhistone substrates. BioMed Res. Int..

[19] Cohen T.J., Guo J.L., Hurtado D.E., Kwong L.K., Mills I.P., Trojanowski J.Q., Lee V.M.Y. (2011). The acetylation of tau inhibits its function and promotes pathological tau aggregation. Nat. Commun..

[20] Noack M., Leyk J., Richter-Landsberg C. (2014). HDAC6 inhibition results in tau acetylation and modulates tau phosphorylation and degradation in oligodendrocytes. Glia.

[21] Kolarova M., García-Sierra F., Bartos A., Ricny J., Ripova D. (2012). Structure and pathology of tau protein in Alzheimer disease. Int. J. Alzheimers Dis..

[22] PÎRŞCoveanu D.F.V., Pirici I., TudoricĂ V., BĂLŞEanu T.A., Albu V.C., Bondari S., Bumbea A.M., PÎRŞCoveanu M. (2017). Tau protein in neurodegenerative diseases—A review. Rom. J. Morphol. Embryol..

[23] Guo T., Noble W., Hanger D.P. (2017). Roles of tau protein in health and disease. Acta Neuropathol..

[24] Reddy P.H. (2011). Abnormal tau, mitochondrial dysfunction, impaired axonal transport of mitochondria, and synaptic deprivation in Alzheimer’s disease. Brain Res..

[25] Brunden K.R., Trojanowski J.Q., Lee V.M.Y. (2008). Evidence that non-fibrillar tau causes pathology linked to neurodegeneration and behavioral impairments. J. Alzheimers Dis..

[26] Lasagna-Reeves C.A., Castillo-Carranza D.L., Sengupta U., Clos A.L., Jackson G.R., Kayed R. (2011). Tau oligomers impair memory and induce synaptic and mitochondrial dysfunction in wild-type mice. Mol. Neurodegener..

[27] Iqbal K., Liu F., Gong C.-X., Alonso A.D.C., Grundke-Iqbal I. (2009). Mechanisms of tau-induced neurodegeneration. Acta Neuropathol..

[28] Ballatore C., Lee V.M.Y., Trojanowski J.Q. (2007). Tau-mediated neurodegeneration in Alzheimer’s disease and related disorders. Nat. Rev. Neurosci..

[29] Mazanetz M.P., Fischer P.M. (2007). Untangling tau hyperphosphorylation in drug design for neurodegenerative diseases. Nat. Rev. Drug Discov..

[30] Irwin D.J., Cohen T.J., Grossman M., Arnold S.E., Xie S.X., Lee V.M.Y., Trojanowski J.Q. (2012). Acetylated tau, a novel pathological signature in Alzheimer’s disease and other tauopathies. Brain.

[31] Irwin D.J., Cohen T.J., Grossman M., Arnold S.E., McCarty-Wood E., Van Deerlin V.M., Lee V.M.-Y., Trojanowski J.Q. (2013). Acetylated tau neuropathology in sporadic and hereditary tauopathies. Am. J. Pathol..

[32] Tracy T.E., Sohn P.D., Minami S.S., Wang C., Min S.W., Li Y., Zhou Y., Le D., Lo I., Ponnusamy R. (2016). Acetylated tau obstructs KIBRA-mediated signaling in synaptic plasticity and promotes tauopathy-related memory loss. Neuron.

[33] Min S.W., Chen X., Tracy T.E., Li Y., Zhou Y., Wang C., Shirakawa K., Minami S.S., Defensor E., Mok S.A. (2015). Critical role of acetylation in tau-mediated neurodegeneration and cognitive deficits. Nat. Med..

[34] Min S.W., Cho S.H., Zhou Y., Schroeder S., Haroutunian V., Seeley W.W., Huang E.J., Shen Y., Masliah E., Mukherjee C. (2010). Acetylation of tau inhibits its degradation and contributes to tauopathy. Neuron.

[35] Rane J.S., Kumari A., Panda D. (2019). An acetylation mimicking mutation, K274Q, in tau imparts neurotoxicity by enhancing tau aggregation and inhibiting tubulin polymerization. Biochem. J..

[36] Tak H., Haque M.M., Kim M.J., Lee J.H., Baik J.-H., Kim Y., Kim D.J., Grailhe R., Kim Y.K. (2013). Bimolecular fluorescence complementation; lighting-up tau-tau interaction in living cells. PLoS ONE.

[37] Lim S., Haque M., Nam G., Ryoo N., Rhim H., Kim Y. (2015). Monitoring of intracellular tau aggregation regulated by OGA/OGT inhibitors. Int. J. Mol. Sci..

[38] Kim D., Lim S., Haque M.M., Ryoo N., Hong H.S., Rhim H., Lee D.E., Chang Y.T., Lee J.S., Cheong E. (2015). Identification of disulfide cross-linked tau dimer responsible for tau propagation. Sci. Rep..

[39] Lim S., Kim D., Ju S., Shin S., Cho I.j., Park S.H., Grailhe R., Lee C., Kim Y.K. (2018). Glioblastoma-secreted soluble CD44 activates tau pathology in the brain. Exp. Mol. Med..

[40] Shin S., Lim S., Jeong H., Kwan L., Kim Y. (2018). Visualization of Tau–Tubulin Interaction in a Living Cell Using Bifluorescence Complementation Technique. Int. J. Mol. Sci..

[41] Liu S.J., Zhang J.Y., Li H.L., Fang Z.Y., Wang Q., Deng H.M., Gong C.X., Grundke-Iqbal I., Iqbal K., Wang J.Z. (2004). Tau becomes a more favorable substrate for GSK-3 when it is prephosphorylated by PKA in rat brain. J. Biol. Chem..

[42] Li Y., Shin D., Kwon S.H. (2013). Histone deacetylase 6 plays a role as a distinct regulator of diverse cellular processes. FEBS J..

[43] Thiagalingam S., CHENG K.H., Lee H.J., Mineva N., Thiagalingam A., Ponte J.F. (2003). Histone deacetylases: Unique players in shaping the epigenetic histone code. Ann. N. Y. Acad. Sci..

[44] Rankin C.A., Sun Q., Gamblin T.C. (2007). Tau phosphorylation by GSK-3β promotes tangle-like filament morphology. Mol. Neurodegener..

[45] Croucher D.R., Iconomou M., Hastings J.F., Kennedy S.P., Han J.Z., Shearer R.F., McKenna J., Wan A., Lau J., Aparicio S. (2016). Bimolecular complementation affinity purification (BiCAP) reveals dimer-specific protein interactions for ERBB2 dimers. Sci. Signal..

[46] Trzeciakiewicz H., Tseng J.H., Wander C.M., Madden V., Tripathy A., Yuan C.X., Cohen T.J. (2017). A dual pathogenic mechanism links tau acetylation to sporadic tauopathy. Sci. Rep..

[47] Ramaiah M.J., Naushad S.M., Lavanya A., Srinivas C., Devi T.A., Sampathkumar S., Dharan D.B., Bhadra M.P. (2017). Scriptaid cause histone deacetylase inhibition and cell cycle arrest in HeLa cancer cells: A study on structural and functional aspects. Gene.

[48] Fleming C.L., Natoli A., Schreuders J., Devlin M., Yoganantharajah P., Gibert Y., Leslie K.G., New E.J., Ashton T.D., Pfeffer F.M. (2019). Highly fluorescent and HDAC6 selective scriptaid analogues. Eur. J. Med. Chem..

[49] Kozikowski A.P., Tapadar S., Luchini D.N., Kim K.H., Billadeau D.D. (2008). Use of the nitrile oxide cycloaddition (NOC) reaction for molecular probe generation: A new class of enzyme selective histone deacetylase inhibitors (HDACIs) showing picomolar activity at HDAC6. J. Med. Chem..

[50] Wang Z.Y., Qin W., Yi F. (2015). Targeting histone deacetylases: Perspectives for epigenetic-based therapy in cardio-cerebrovascular disease. J. Geriatr. Cardiol..

[51] Lakshmaiah K., Jacob L.A., Aparna S., Lokanatha D., Saldanha S.C. (2014). Epigenetic therapy of cancer with histone deacetylase inhibitors. J. Cancer Res. Ther..

[52] Carlomagno Y., Chung D.E.C., Yue M., Castanedes-Casey M., Madden B.J., Dunmore J., Tong J., DeTure M., Dickson D.W., Petrucelli L. (2017). An acetylation–phosphorylation switch that regulates tau aggregation propensity and function. J. Biol. Chem..

[53] Tseng J.H., Xie L., Song S., Xie Y., Allen L., Ajit D., Hong J.S., Chen X., Meeker R.B., Cohen T.J. (2017). The Deacetylase HDAC6 mediates endogenous Neuritic tau pathology. Cell Rep..

[54] Lu X., Wang L., Yu C., Yu D., Yu G. (2015). Histone acetylation modifiers in the pathogenesis of Alzheimer’s disease. Front. Cell. Neurosci..

[55] Bahari-Javan S., Sananbenesi F., Fischer A. (2014). Histone-acetylation: A link between Alzheimer’s disease and post-traumatic stress disorder?. Front. Neurosci..

[56] Gräff J., Rei D., Guan J.-S., Wang W.-Y., Seo J., Hennig K.M., Nieland T.J., Fass D.M., Kao P.F., Kahn M. (2012). An epigenetic blockade of cognitive functions in the neurodegenerating brain. Nature.

