# Supplementary material for: Pan-HDAC Inhibitors Promote Tau Aggregation by Increasing the Level of Acetylated Tau
Source: Int J Mol Sci. 2019 Sep 1;20(17):4283. doi: 10.3390/ijms20174283 (PMC6747090; doi:10.3390/ijms20174283)
Supplement: Supplementary file 1 [file ijms-20-04283-s001.pdf]

Supplementary Information

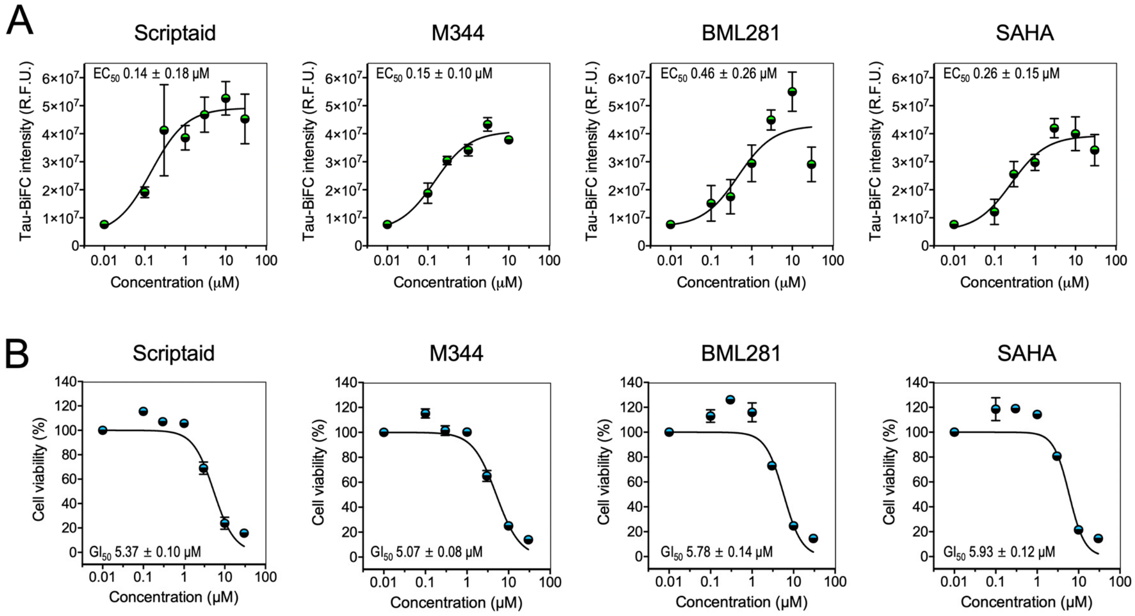

Figure S1. Dose-dependent analysis of Scriptaid, M344, BML281 and SAHA. (**A**,**B**) Tau-BiFC cells were incubated with Scriptaid, M344, BML281, or SAHA at various concentrations (0.1, 0.3, 1, 3, 10, 30 μM) for 36 h. Nuclei were counterstained with Hoechst. BiFC fluorescence intensities were quantified using Harmony 3.1 software. A Prism's non-linear regression analysis was used to measure the EC_{50} and GI_{50} values. The EC_{50} represents the concentration of a compound that gives half-maximal tau-BiFC response. The GI_{50} represents the concentration of a compound that reduces total cell growth by 50%.
